# Tannin-Based Nanoscale Carbon Spherogels as Electrodes for Electrochemical Applications

**DOI:** 10.1021/acsanm.1c03431

**Published:** 2021-12-02

**Authors:** Ann-Kathrin Koopmann, Jorge Torres-Rodríguez, Miralem Salihovic, Juergen Schoiber, Maurizio Musso, Gerhard Fritz-Popovski, Nicola Huesing, Michael S. Elsaesser

**Affiliations:** † Department of Chemistry and Physics of Materials, Paris-Lodron-University of Salzburg, 5020 Salzburg, Austria; ‡ Salzburg Center for Smart Materials, 5020 Salzburg, Austria; § Institute of Physics, Montanuniversitaet Leoben, 8700 Leoben, Austria

**Keywords:** aerogels, sustainable carbons, nanoporous materials, hollow carbon spheres, electrochemistry, tannin

## Abstract

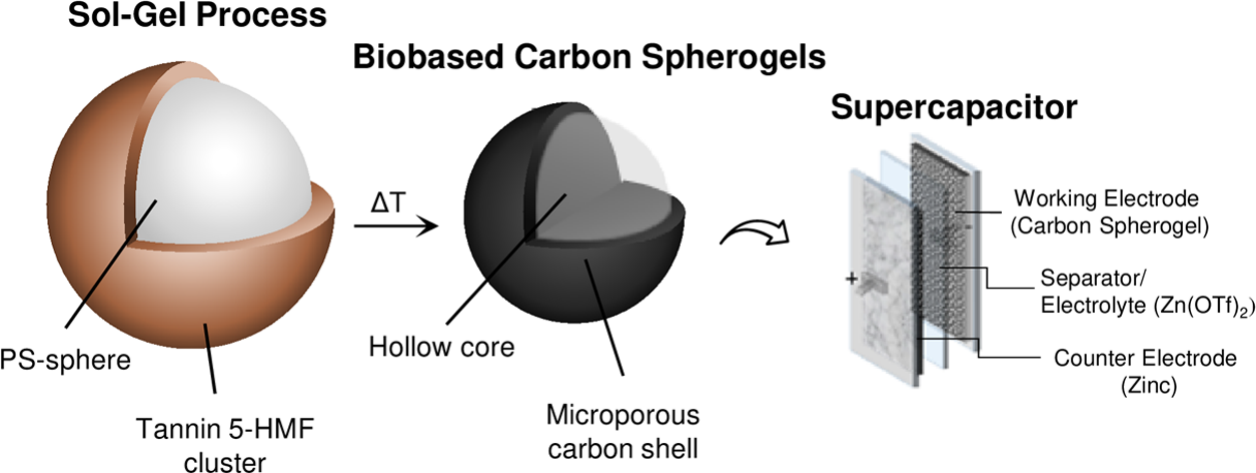

A promising route to monolithic, hollow sphere carbon assemblies based on sustainable precursors with a tailored nanostructure is presented. These carbon assemblies, recently termed carbon spherogels, are generated via a polystyrene sphere template-based sol-gel process of mimosa tannin and biomass-derived 5-(hydroxymethyl)furfural. By completely replacing petroleum-based precursors (especially toxic formaldehyde) highly porous, nanoscale carbon monoliths are obtained, which are investigated as state-of-the-art, sustainable electrode materials for energy storage. This study defines the required synthesis parameters, in particular the highly acidic initial pH and a tannin/water ratio of at least 0.05 or lower, for a successful and homogeneous generation of these biobased carbon spherogels.

## Introduction

Nowadays, the development of energy storage devices, which are based on sustainable, green, and non-toxic materials, is of crucial demand for a future society, in which the impact of carbon dioxide on nature must be drastically reduced.^1^ Carbon aerogels are promising candidates for energy storage materials, since they consist of a highly porous network, featuring specific surface areas up to 2500 m^2^ g^–1^, and electrical conductivity.^2^ The synthesis route to carbon aerogels typically comprises three steps: (1) formation of an organic aqua gel via sol-gel processing, (2) supercritical drying to obtain an organic aerogel, and (3) carbonization in an inert gas atmosphere.^3^ Besides, using sacrificial, spherical templates during sol-gel processing represents an effective method to tailor the nanoscale morphology of carbon aerogels.^4^ This strategy results in homogeneous, hollow carbon sphere structures. Such hollow carbon spheres are mainly generated by either hard-,^5−7^ soft-,^8,9^ or self-templating^10,11^ in combination with different preparation techniques, such as chemical vapor deposition, sol-gel processing, spray pyrolysis, selective etching, and many more.^12^ To name a specific method, polystyrene (PS) sphere templating is conveniently applied, which allows the generation of hollow carbon sphere powders,^13−15^ mesocellular carbon foams,^16^ and monolithic gels, the so-called carbon spherogels.^17,18^ Overall, such monolithic, hollow sphere carbon materials have gained increased interest, as they feature the possibility of precise control of the pore structure, a high surface-to-volume ratio, and an interior void space suitable for encapsulation.^12,14,19,20^

A variety of organic systems (e.g., resorcinol–formaldehyde,^15,21−23^ mesophase pitch,^24^ graphene,^25^ and carbon nanotubes^26^) have been explored as carbon precursors. However, these approaches are all based on petroleum-derived precursors, thus limiting the eco-friendliness of these materials. However, the demand for green, sustainable carbon materials for industrial applications is increasing over the past years. Thus, biomass-derived biomolecules, such as carbohydrates, chitin, proteins, and tannins have been investigated regarding their suitability as a carbon precursor source.^1,27^ In particular, with regard to the generation of sustainable hollow carbon materials, the usage of mono- and polysaccharides was studied.^13,14^ Generally, these biomass-derived carbons are generated via different methods, namely, hydrothermal carbonization, pyrolysis, mechanochemical synthesis, hard- or soft-templating.^27^ On the way to sustainably- sourced carbons, intensive research is particularly devoted to the (partial) replacement of non-green precursors by wood-based reagents, such as tannin or lignin, with formaldehyde as a crosslinker.^28−30^ These renewable precursors are available at low cost in a megaton scale or even as waste products from the paper/wood industry.^31^ However, the aforementioned materials still require the use of toxic formaldehyde as a crosslinker. Hence, an alternative biofriendly crosslinker has to be found to obtain a fully sustainable class of aerogels. Recently, some efforts have been made replacing formaldehyde by other reagents such as glyoxal, glutaraldehyde, and hexamethylenetetramine. These reagents already display less toxic and environmentally harmful properties, but they however show weaker crosslinking efficiency than formaldehyde.^32^ Thus, research for “green” alternatives to formaldehyde is still ongoing. Therefore, in regard to tannin wood adhesives, the biomass-derived 5-(hydroxymethyl)furfural (5-HMF)^33−35^ has been investigated.^36^ The particular interest in this aldehyde derivate of furan arises due to the two reactive functions of the molecules, namely, its aldehyde group as well as the reactive hydroxymethyl group, present on the furan ring.^36^

In this study, a route to generate freestanding, monolithic, nanoscale hollow carbon materials by using green, sustainable precursors, which replace the commonly applied petroleum-derived ones, such as toxic resorcinol–formaldehyde, is presented. Thus, the preparation of carbon spherogels based on the PS templating approach using the organic precursor mimosa tannin, which can be obtained by simple extraction from bark, and the biomass-derived crosslinker 5-HMF is discussed. Furthermore, the suitability of these carbon spherogels for use as electrodes in electrochemical applications will be investigated and compared to the performance of state-of-the-art carbons.

## Experimental Section

### Chemicals

The commercially available mimosa tannin “Weibull” extract was acquired from Tanac Company (Brazil). 5-HMF (≥95%) was supplied by AVA Biochem (Switzerland). Technical grade acetone (>99%) and sodium hydroxide pellets were provided by VWR. Hydrochloric acid (37%) was supplied by Merck. Styrene (≥99%), zinc trifluoromethanesulfonate (Zn(OTf)_2_, 98%), sodium carboxymethyl cellulose (Na-CMC, 99%), and polyvinylpyrrolidone (average mol wt 40,000) were acquired from Sigma-Aldrich. Potassium persulfate was supplied by Honeywell Fluka. Styrene–butadiene–rubber (SBR) suspension was gifted by ZEON Europe GmbH. Zinc foil (99.9%) and glass fiber filters (GF\B, Whatman) were purchased from Alfa Aesar.

### Synthesis of Carbon Spherogels

The PS spheres were synthesized, according to Du and He,^37^ by an emulsion polymerization reaction of styrene with potassium persulfate as the initiator and polyvinylpyrrolidone as the stabilization agent. The obtained PS sphere solution (average diameter: 213.0 ± 3.5 nm, Figure S1) was diluted to a final concentration of 3, 6, 9, or 12 wt %. This aqueous PS solution was used as a templating agent for the generation of carbon spherogels. For the generation of the organic gels, 0.31 g of mimosa tannin and 5.68 g of the aqueous PS solution (3 wt %) were mixed. Afterward, 0.63 g (0.5 mmol) of the crosslinker 5-HMF was added. Then, the pH of the suspension was adjusted to a certain value using 1 M HCl or 0.1 M NaOH, whereas the dilution of the solution due to adjusting the pH is negligible. After further stirring, the solution was filled inside glass vessels and placed in an oven at 80 °C to promote gelation. After 7 days of aging, the gels were liberated from the glass vials and stored in 1 M HCl solution. After 24 h in the HCl solution, the gels were subjected to solvent exchange using acetone (24 h cycle, five times, 50 mL) to ensure complete solvent exchange and extraction of the non-reacted species and byproducts. Then, the wet gels were dried using supercritical extraction with CO_2_ (110 bar, 60 °C). The obtained organic gels were carbonized in a tube furnace (800 °C, 60 °C h^–1^, 2 h, 75 NL h^–1^ argon flow). Prior to the electrochemical characterization, the carbonized spherogels were physically activated with CO_2_ in a tube furnace (800 °C, 600 °C h^–1^, 4–6 h, 1 NL min^–1^ CO_2_ flow).

The specific amounts of employed precursors for the generation of the sustainable carbon spherogel samples and the nomenclature of the samples are listed in the Supporting Information (Table S1 and Figure S2, respectively).

### Materials Characterization

The morphology of carbon spherogels was analyzed by scanning electron microscopy (SEM), recorded with a Zeiss Ultra Plus instrument, using an in-lens secondary electron detector as well as a varying acceleration voltage (5–10 kV). Transmission electron microscopy (TEM) images were taken with a JEOL JEM F200 microscope, which is equipped with a cold field emission source and uses a TVIPS F216 2k × 2k CMOS camera. In general, the TEM images were obtained using an electron acceleration voltage of 200 keV.

Nitrogen sorption isotherms were recorded on a Sy-Lab Micromeritics ASAP 2420 surface area and porosity analyzer at −196 °C and in a relative pressure range *p*/*p*_0_ from 10^–7^ to 1. Prior to analysis, the samples were degassed at 300 °C for 12 h under vacuum. The obtained isotherms were analyzed using the MicroActive (Version 5.0) software. The usage of the non-local density functional theory (NLDFT), in particular utilizing the method N2@77 on Carbon Slit Pores by NLDFT, allows the determination of specific surface area (SSA) and pore size distribution (PSD) of the samples.

Raman spectra were collected within the range of 500–3500 cm^–1^ with a 532 nm laser excitation wavelength and a laser power of 4 mW. A Thermo Scientific DXR2 Raman microscope was used, which was equipped with a confocal microscope BX41 (Olympus Corp.) and a 10× objective, delivering a laser spot diameter of approximately 2 μm, allowing a spectral resolution (equivalent to the full width at half-maximum of the instrumental line width) of about 8 cm^–1^ with a 50 μm pinhole-like entrance slit to the spectrometer.

Small-angle X-ray scattering (SAXS) curves were measured using a Nanostar (Bruker AXS), which was equipped with an IμS microsource (Incoatec), 300 μm SCATEX pinholes (Incoatec), and a VÅNTEC-2000 detector (Bruker AXS). The radiation used was Cu Kα. Sample–detector distances were determined by silver behenate calibration and found to be 1102 and 319 mm, which allowed for an angular range of 0.08 < *q* < 8 nm^–1^, where *q* = 4·π/λ·sin(ϑ/2), λ is the wavelength, and ϑ is the scattering angle. Measurements were repeated five times and averaged. A transmission-corrected background was subtracted. Data were evaluated using indirect Fourier transformation^38^ and model approximation.^18^

Thermogravimetric analysis (TGA) was carried out on a Netzsch STA 449 F3 Jupiter instrument. The samples were heated at a rate of 10 °C min^–1^ from 20 to 1000 °C under argon atmosphere.

The size and surface potential of the PS spheres were analyzed by dynamic light scattering (DLS) and zeta potential measurements, which were performed on a Malvern Zetasizer instrument. Each measurement consisted of three separate DLS measurements with 30 sub-runs each.

### Electrochemical Properties

Selected synthesized carbon spherogels were tested as supercapacitor materials against Zn/Zn^2+^ in a self-made cell. The carbon materials were ground in a glass vessel with zirconia balls while vortexing before electrode preparation. The weight ratios were chosen for the electrode preparation as 90 wt % of the electroactive material and 10 wt % binder. In particular, 18 mg of a carbon spherogel sample was suspended in an aqueous binder solution (2 mg of binder mixture in 180 μL of water), consisting of one part of Na-CMC and three of parts SBR (0.5 mg of Na-CMC and 1.5 mg of SBR), through ultrasound treatment. The prepared slurry was drop-casted on a titanium current collector, with a geometric surface of 1.3 cm^2^, targeting a mass load between 1.0 and 1.5 mg cm^–2^, and dried for 1 h at 80 °C in a vacuum oven. Whatman glass fibers as the separator, an aqueous 3 mol kg^–1^ Zn(OTf)_2_ solution as the electrolyte, and a disc of metallic zinc foil as counter and reference electrodes were used for the electrochemical cell setup. The supercaps were assembled under ambient conditions and electrochemically tested using a single-channel BioLogic SP-50 potentiostat. The storage behavior based on the mass load of the active material was determined by galvanostatic charge/discharge cycling with constant currents. All galvanostatic measurements were carried out in the voltage range of 0.0–1.9 V (vs Zn/Zn^2+^) at current densities between 0.5 and 20 A g^–1^, except for long-term cycling tests (4000 cycles at 8 A g^–1^). Cyclic voltammetry (CV) measurements were performed in the voltage range of 0.0–1.9 V (vs Zn/Zn^2+^) with sweep rates from 1 to 20 mV s^–1^. All measurements were carried out at ambient conditions.

The specific capacitance has been calculated from charge/discharge processes according to the equation^39^
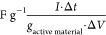
1where *I* is the applied current in amperes, Δ*t* in seconds is the time interval for the discharge process, *g*_active material_ is the mass in grams of the active material, and Δ*V* is the potential interval in volts.

## Results and Discussion

**Figure 1:**
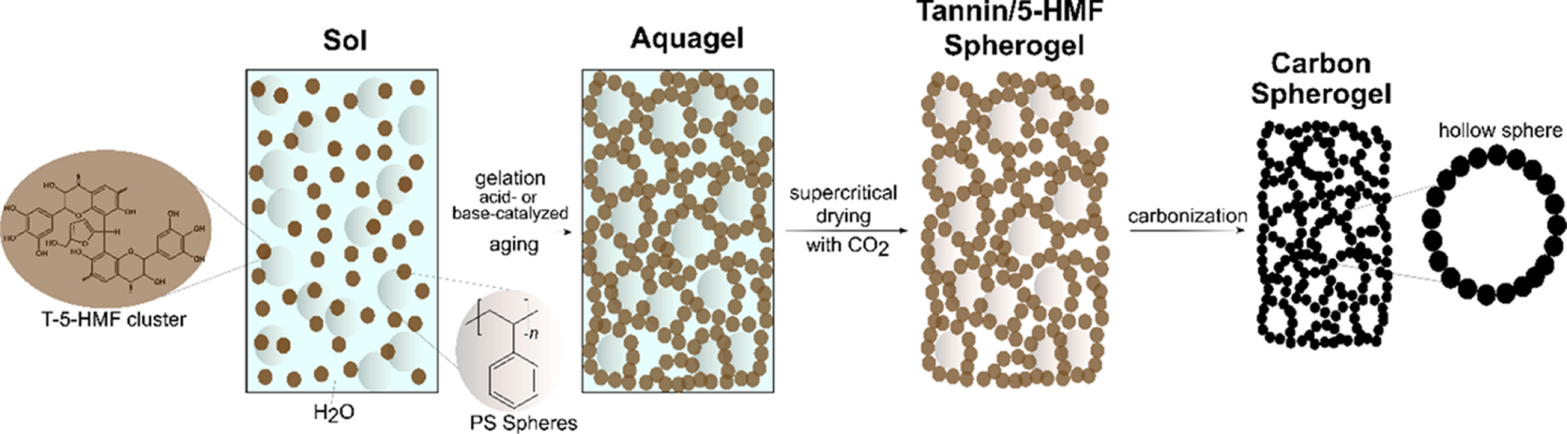
Reaction scheme illustrating the generation of GCSs using a PS templating approach with the tree extract mimosa tannin and the biobased 5-HMF as the carbon source.

The generation of petroleum-derived carbon spherogels has already been well discussed.^17,18^ However, targeting the sustainability of these kinds of carbon materials, the usage of the tree extract mimosa tannin (T), which is a low-cost waste product from the paper industry, and the biomass-derived 5-HMF has been investigated as a suitable carbon source. Hence, the synthesis of green carbon spherogels (GCSs), based on sol-gel processing of these sustainable precursors in combination with PS sphere templating, was systematically investigated and optimized. A schematic pathway of our strategy is illustrated in Figure 1: In the first step, an organic, monolithic aquagel is formed via condensation of tannin with 5-HMF, whereby a covalently connected T-5-HMF cluster is formed via methylene bridges, as proposed by Santiago-Medina and co-workers.^36^ By using the sphere-templating method, this T-5-HMF network cluster, simultaneously to its network formation, coats the PS spheres by ionic interactions with the negatively charged surface of the spheres (Supporting Information, Figure S3). During the aging process, further inter-particular coalescence occurs. Subsequently, solvent exchange was accomplished first in HCl to strengthen the gel network, followed by exchange to acetone to allow supercritical drying with carbon dioxide. Supercritical drying is applied to minimize shrinkage effects. Finally, by carbonization at 800 °C under an argon atmosphere, the PS sphere templates are decomposed, as shown by TGA (Supporting Information, Figure S4), and a carbon spherogel monolith, solely composed of hollow carbon spheres, is obtained. The carbonization process results in minor linear shrinkage of the monoliths accounting 20%.

Various synthesis parameters (theoretical density, tannin/water ratio, pH value, and PS concentration) determining whether carbon spherogel morphologies or rather aerogel-related structures comprised a nanoparticle network are obtained. The successful generation of GCSs hence requires a careful control of the process parameters. In particular, the theoretical density and accordingly the tannin/water ratio (T/W) as well as the initial pH value of the solution represent the critical parameters with respect to the morphology of the resultant materials and are hereinafter discussed.

### Tannin/Water Ratio

**Figure 2:**
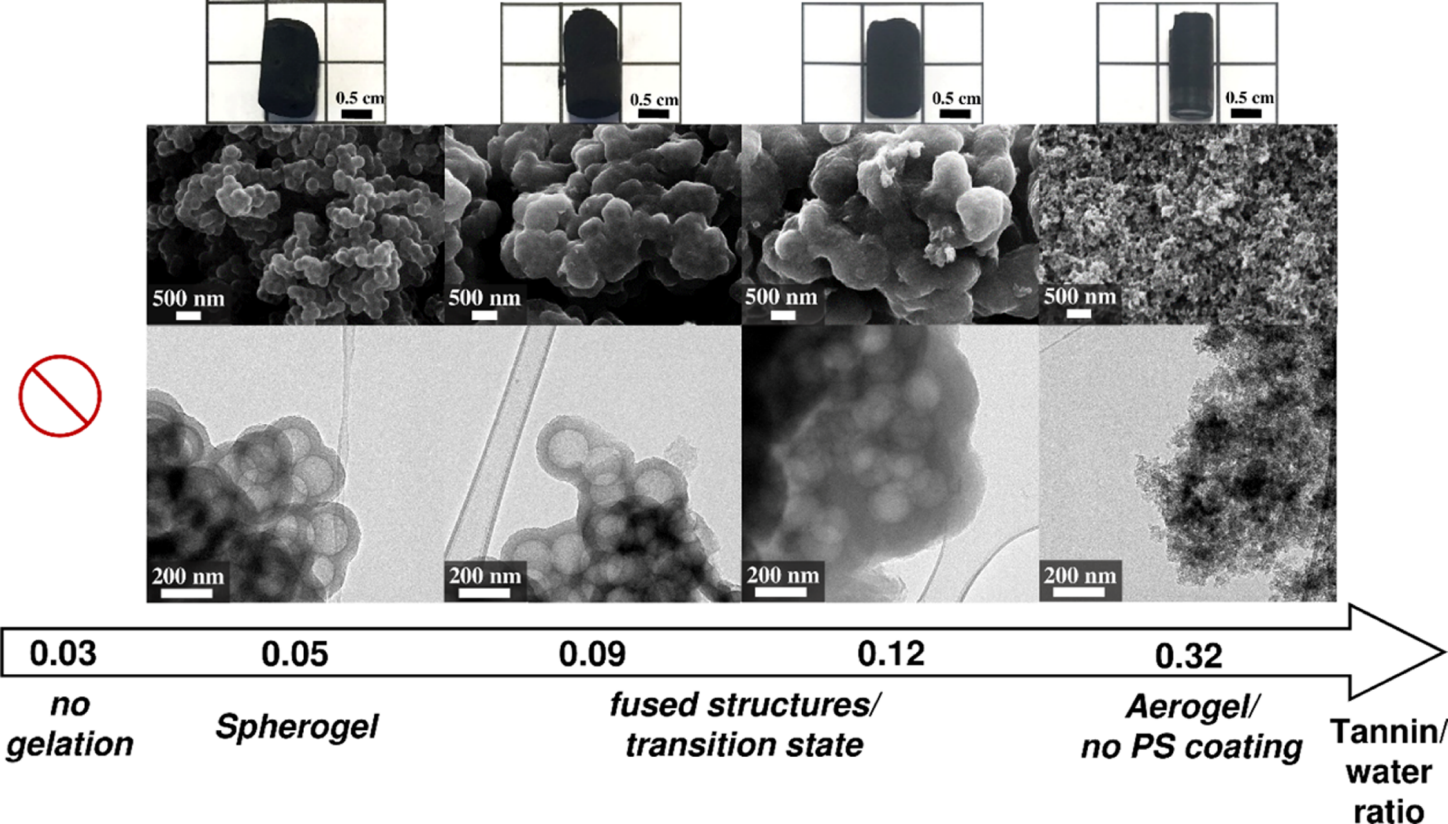
GCS structures synthesized at a fixed pH value of 3, a PS concentration of 3 wt %, a T/5-HMF ratio of 0.5 but with varying tannin/water ratios (different theoretical densities) visualized by SEM and TEM images. Digital photographs of the carbon monoliths are given in the top row.

The T/W ratio not only defines the theoretical density of the formed monoliths, but also represents a decisive parameter for the generation of a gel network solely consisting of hollow carbon spheres. This T/W dependency on the obtained gel network morphologies after carbonization is illustrated by the TEM images in Figure 2. The T/W ratio is varied in the range of 0.03–0.32 (theoretical density: 0.025–0.2 g cm^–3^) while keeping the T/5-HMF ratio (0.5), the sphere templating concentration (3 wt %), and the initial pH value (3.0) constant. A high dilution of tannin of T/W = 0.03 (lowest investigated theoretical density: 0.025 g cm^–3^) of the tannin-5-HMF precursor sol induces no gelation. Thus, presumably the low tannin content in correspondence to the aqueous media does not lead to a sufficient coating of the PS spheres, hence resulting in a weakly or not interconnected tannin network. In contrast, the lowest dilution of T/W = 0.32 (highest investigated theoretical density: 0.2 g cm^–3^) yields only a conventional carbon aerogel network with no coating of the PS spheres. In between these dilution and saturation limits, a certain window exists, where the generation of carbon spherogels is possible. However, depending on the degree of dilution, an influence on the network generation of carbon spherogels can be observed. Precisely, a pure spherogel network is produced at a T/W ratio of 0.05 (theoretical density: 0.05 g cm^–3^), similar to the resol-based spherogels (R/W weight ratio = 0.05).^18^ However, slightly higher T/W ratios of 0.09 and 0.12 (slightly lower dilutions; theoretical densities: 0.075 and 0.1 g cm^–3^, respectively) already yield a mixed morphology (transition state), which comprised a partial spherogel network, together with a carbon nanoparticle aerogel structure.

### Initial pH Value

**Figure 3:**
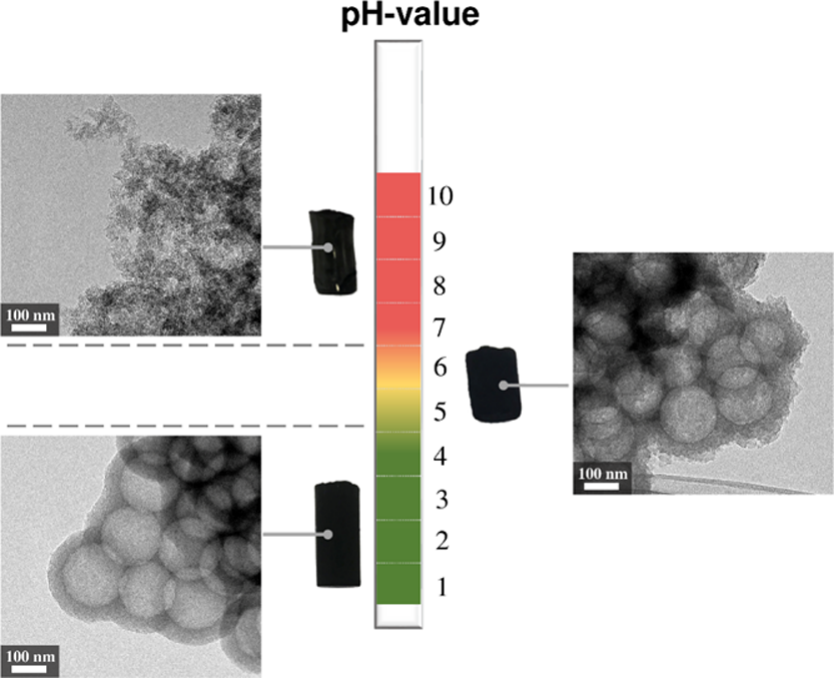
Representation of the pH dependence (pH 2, 5, and 8) on the spherogel structure at a given tannin/water ratio of 0.05 and a T/5-HMF ratio of 0.5, visualized by transmission electron micrographs.

In order to investigate the influence of the pH, several carbon gels with a T/W ratio of 0.05, a T/5-HMF ratio of 0.5, and a PS concentration of 3 wt %, but with different pH values, ranging from 1–10, were synthesized. Three representative structures obtained for different pH values (2, 5, and 8) are shown in the transmission electron micrographs in Figure 3. In particular, if the reaction proceeds under highly acidic conditions (pH < 4), pure carbon spherogels are obtained without the evidence of the typical particulate aerogel nanostructure. By further increasing the pH value (pH 4–6), a transition state is reached, wherein an amorphous tannin network around the carbonized PS spheres is formed in a non-defined manner. This suggests the partial deprotonation of hydroxyl groups of tannins. At higher pH values (pH > 6), tannins are entirely deprotonated and hence show repulsion by the negatively charged surface of the PS spheres, yielding a carbon aerogel network, composed of carbon nanoparticles. The full spectrum of morphologies, visualized by scanning electron micrographs, from pH 1–10 is shown in the Supporting Information, Figure S5.

**Figure 4:**
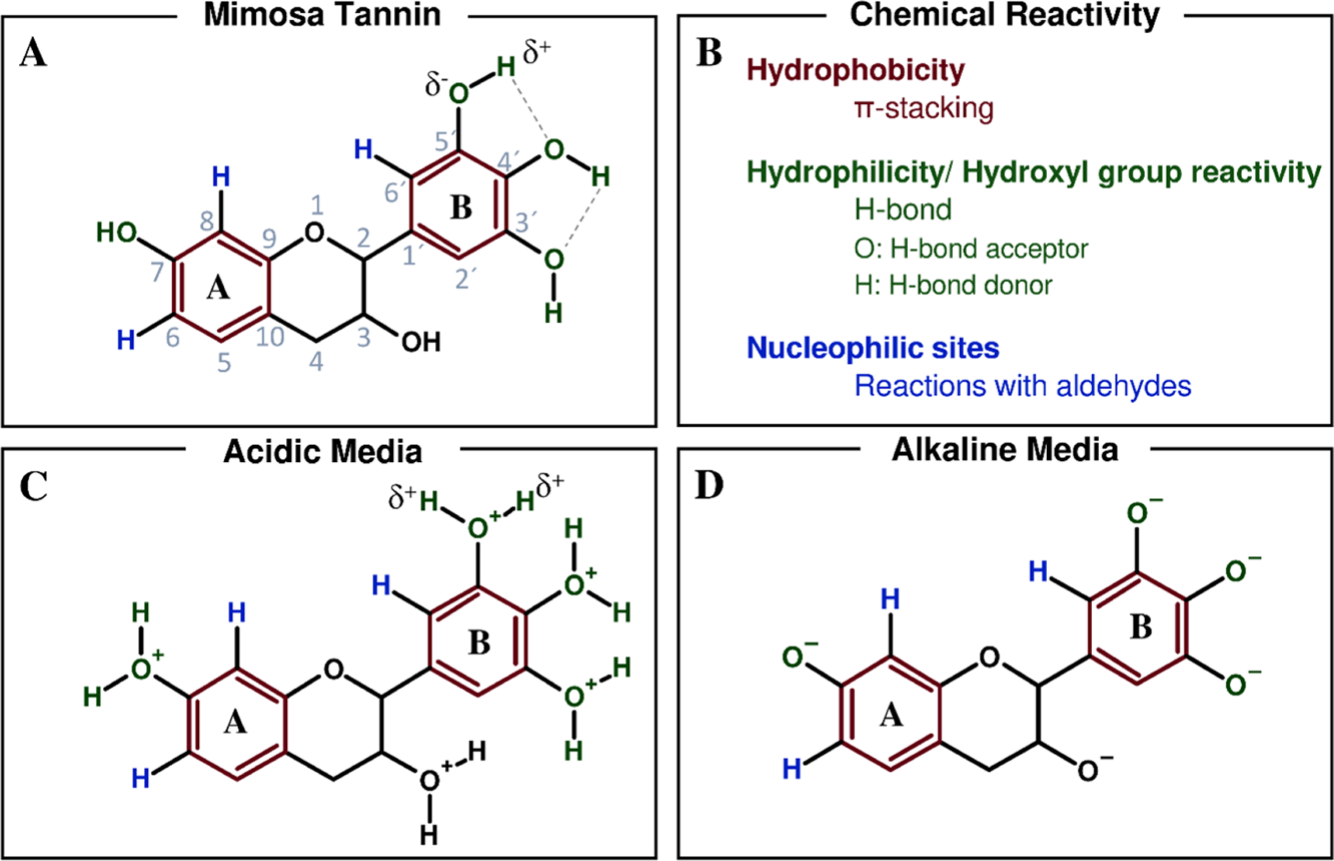
Chemical structures of the prorobinetinidin unit of mimosa tannin (A) and a list of possible chemical reactions (B) and structures in acidic media (C) and alkaline media (D), whereas the degree of protonation and deprotonation depicts a function of pH.

Precisely, the impact of the initial pH value on network formation can be explained by the chemical state of the prorobinetinidin unit, the main representative in mimosa tannin, visualized in Figure 4A, as well as its chemical reactivity (Figure 4B). Generally, the spherogel network formation is fostered by π-stacking attracting intermolecular interactions between styrene and tannins’ aromatic rings.^40,41^ Furthermore, the synthesized PS spheres exhibit a negative surface charge of −42.7 mV (Supporting Information, Figure S3B) due to the presence of sulfate groups, which are derived from the initiator potassium persulfate during the PS synthesis. Thus, in addition to the π-stacking, the hydroxyl groups of tannins presumably show ionic interactions to the sulfate groups on the surface of PS spheres. As a consequence, a homogeneous tannin network coating around the PS spheres is formed. However, this coating mechanism is highly dependent on the protonation/deprotonation degree of the hydroxyl groups of tannins (average p*K*a value: 9.3) in acidic or alkaline media (Figure 4C,D), respectively.^42^ Precisely, at high pH values, deprotonation of the hydroxyl groups of tannins takes place, resulting in the repulsion of tannins from the negatively charged PS spheres. On the contrary, highly acid pH values prevent the deprotonation of tannins and even trigger the attraction of tannins and PS spheres due to the (partial) protonation of hydroxyl groups of tannins.

### Effect of the Concentration of PS Spheres

In virtue of the above findings, a T/W ratio of 0.05 and an initial pH value of 1 were chosen to further investigate the spherogel formation with regard to the applied template concentration. Different amounts of PS spheres as templates offer on the one side higher or lower surface area for gelation, and on the other side, after template removal, the overall hollow sphere interior volume can be adjusted.^18^ In a series with varying PS concentrations from 3 to 12 wt %, monolithic carbon spherogel samples were prepared. These synthesized GCSs were termed according to the used PS concentration, for example, GCS_3, when a PS concentration of 3 wt % was applied. Furthermore, activated samples are labeled with the addition of _6a (Supporting Information, Figure S2).

Using Raman spectroscopy, the structure of the non-activated carbon spherogels has been investigated, and the obtained Raman spectra are visualized in Supporting Information Figure S6. Each of the spectra shows the distinct D-band at about 1337 cm^–1^ and G-band at roughly 1588 cm^–1^, which correspond to the sp^2^-hybridized disordered carbon (A_1g_ in-plane breathing vibration mode) and ordered graphite (E_2g_ in-plane vibration mode), respectively.^43^ In general, the D-band is not visible in pure graphitic structure but becomes more visible with a more disordered carbon structure. Hence, the ratio between the areas of the D- and G-bands (*A*_D_/*A*_G_ ratio) characterizes the relative degree of graphitization of the carbon material, with a higher ratio indicating a more disordered structure.^43,44^ All carbon spherogels, generated at different PS concentrations, feature a peak area ratio (*A*_D_/*A*_G_) of approximately 2.4 and thus reveal an incomplete crystalline characteristic with a large contribution of amorphous carbon, similar to the petroleum-based carbon spherogels as well as other resol-based carbon aerogels.^17,18,45,46^ Furthermore, the disordered carbon structure is also indicated by a broadening of the D- and G-bands. Hence, these bands are cumulatively fitted by deconvolution into five bands, namely, D*, D, D**, G, and D′, according to Kaniyoor and Ramaprabhu (Supporting Information, Figure S7).^47^ Next to the conventional D- and G-bands, D* and D**, observed at roughly 1310 and 1550 cm^–1^, respectively, also show a great contribution and thus indicate, similar to the *A*_D_/*A*_G_ ratio, a disordered amorphous carbon material.^47^

**Figure 5:**
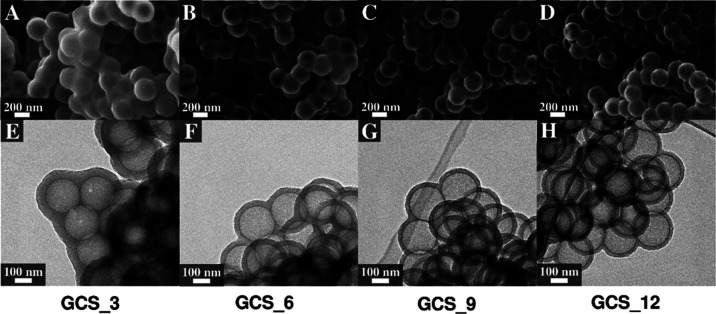
Scanning electron micrographs (A–D) and corresponding transmission electron micrographs (E–H) of carbon spherogels, which were templated with 3, 6, 9, and 12 wt % PS solution, respectively.

Figure 5 represents the microstructural variations of these spherogels, analyzed by SEM as well as TEM. According to the SEM images (Figure 5A–D), the single spheres are fused to their adjacent spheres at lower PS concentrations. On the opposite, they become well isolated with an increasing amount of PS template. The TEM images (Figure 5E–H) reveal the homogeneous morphology of solely hollow spheres and furthermore a direct dependency of the sphere wall thickness related to the applied template concentration. More precisely, the wall thickness of the spheres decreases with an increasing PS concentration at an equal amount of tannin/5-HMF (Table 1), as already demonstrated in studies regarding petroleum-based spherogels.^18^ In detail, the wall thickness varies from 47.1 nm (±1.8 nm) for a low 3 wt % PS concentration down to a wall thickness of 39.7 nm (±1.0 nm), 32.1 nm (±1.6 nm), and 21.1 nm (±1.2 nm) for a higher 6, 9, or 12 wt % PS concentration, respectively. On the contrary, the diameter of the hollow cores remains constant at roughly 193 nm for all the synthesized spherogels (Table 1).

**Table 1:** Physical Properties, Obtained from Nitrogen Sorption Analysis, SAXS Measurements, and TEM Images, of Carbon Spherogels, Templated at Different PS Concentrations before and after Activation

	SSA (NLDFT) (m^2^ g^–1^)		specific pore volume^a^ (NLDFT) (cm^3^ g^–1^)		micropore volume^b^ (NLDFT) (cm^3^ g^–1^)		inner diameter^c^ (TEM) (nm)	wall thickness^c^ (TEM) (nm)	wall thickness (SAXS) (nm)	micropore diameter (SAXS) (nm)	bulk density ρbulk (g cm^–3^)
sample	synthesized	activated	synthesized	activated	synthesized	activated	synthesized	synthesized	synthesized	synthesized	synthesized
GCS_3	850	1894	0.35	0.68	0.30	0.63	192 ± 3	47.1 ± 1.8	46.8	0.36	0.08 ± 0.01
GCS_6	861	1898	0.44	0.80	0.34	0.69	194 ± 3	39.7 ± 1.0	37.6	0.38	0.07 ± 0.01
GCS_9	895	1904	0.43	0.82	0.35	0.71	193 ± 2	32.1 ± 1.6	35.6	0.37	0.07 ± 0.01
GCS_12	851	1971	0.59	0.76	0.39	0.68	192 ± 2	21.1 ± 1.2	24.6	0.40	0.07 ± 0.01

a ≤30 nm.

b ≤2 nm.

c The inner diameter as well as the wall thickness was determined by measuring five spheres in transmission electron scanning micrographs using the EM Measure software.

All carbon spherogels with different amounts of PS template reveal very low bulk densities in the range of 0.07–0.08 g cm^–3^, which are only negligibly higher compared to the resol-based spherogels, featuring a bulk density of 0.06 g cm^–3^.^18^ As visualized by the TEM micrographs in Figure 5E–H, a macroporous space is present between the hollow sphere assemblies.

**Figure 6:**
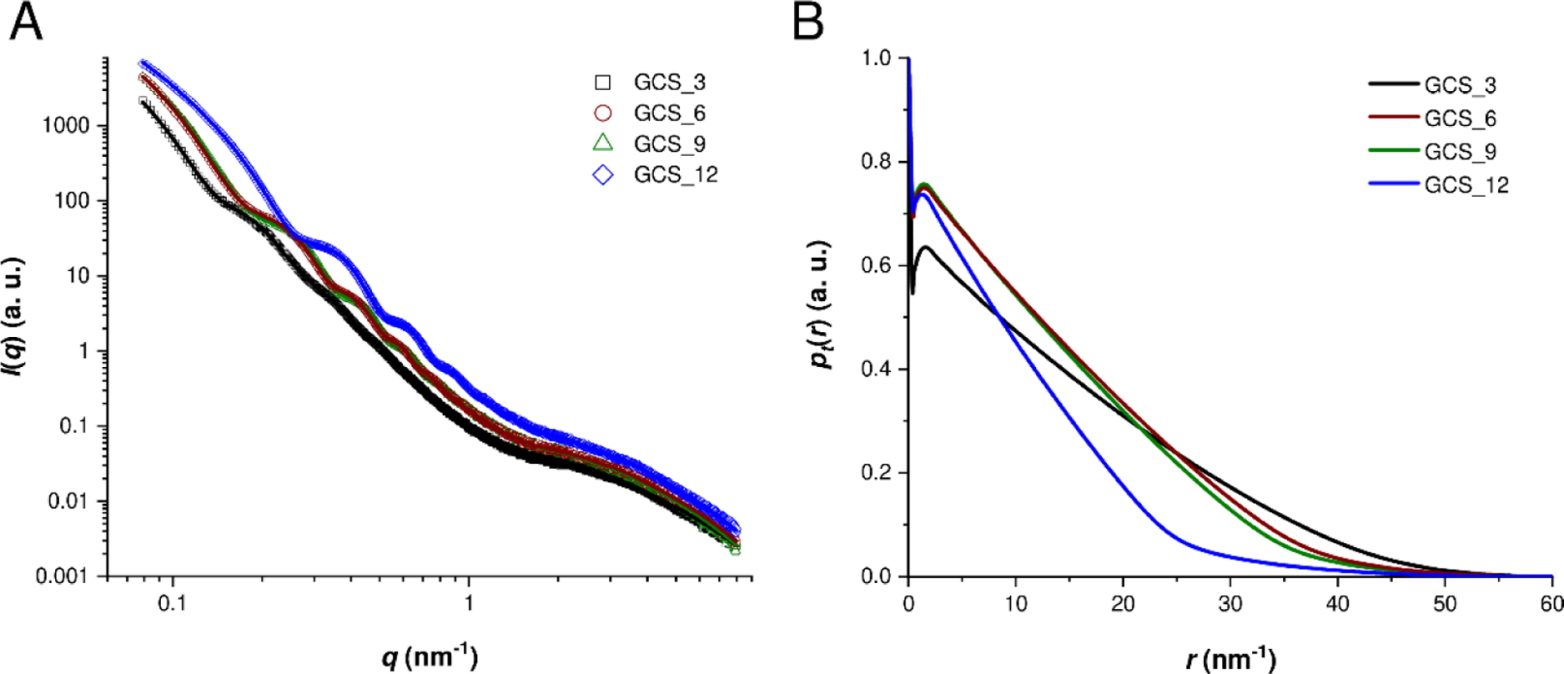
SAXS curves (symbols) and their fits (line) (A) as well as the resulting real space curves (B) of the carbon spherogels GCS_3 (black), GCS_6 (red), GCS_9 (green), and GCS_12 (blue).

The SAXS curves (Figure 6A) show decay at small angles, oscillations at intermediate angles, and a shoulder at large angles. The decay at low angles is due to the overall structure, which is beyond the accessible size range of the instrument. However, the GCS_12 sample shows that this decay corresponds to a *q*^–2^-power law, which in turn corresponds to locally flat structures. This is due to the fact that the curvature of the shell is small, resulting in small effects on the length scale probed by SAXS. The signal is caused by the shell that can therefore be interpreted as that of flat structures with negligible contributions by the curvature. The oscillations hide the *q*^–2^-power law for the other samples. The oscillations can be interpreted as to be caused by flat structures, and their positions are indirectly proportional to the thicknesses of the shells (Table 1). Higher order minima are found, indicating a low polydispersity of wall thickness especially for samples with high PS concentrations. The imperfect separation of total structure and locally flat shell prevents quantification of the polydispersity. Finally, the shoulder at large angles corresponds to micropores that have a diameter of about 0.4 nm (Table 1). These findings are corroborated by an indirect Fourier transformation of the scattering curves assuming flat structures. The resulting real space curves (Figure 6B) show a strong peak at small distances up to 0.4 nm, which is due to the micropores. Distances above 2 nm show a linear decay, whose extrapolation crosses the abscissa at a distance similar to the shell thickness. The deviations at large distances from the linear decay are due to the deviations of the shell from a truly flat structure.

**Figure 7:**
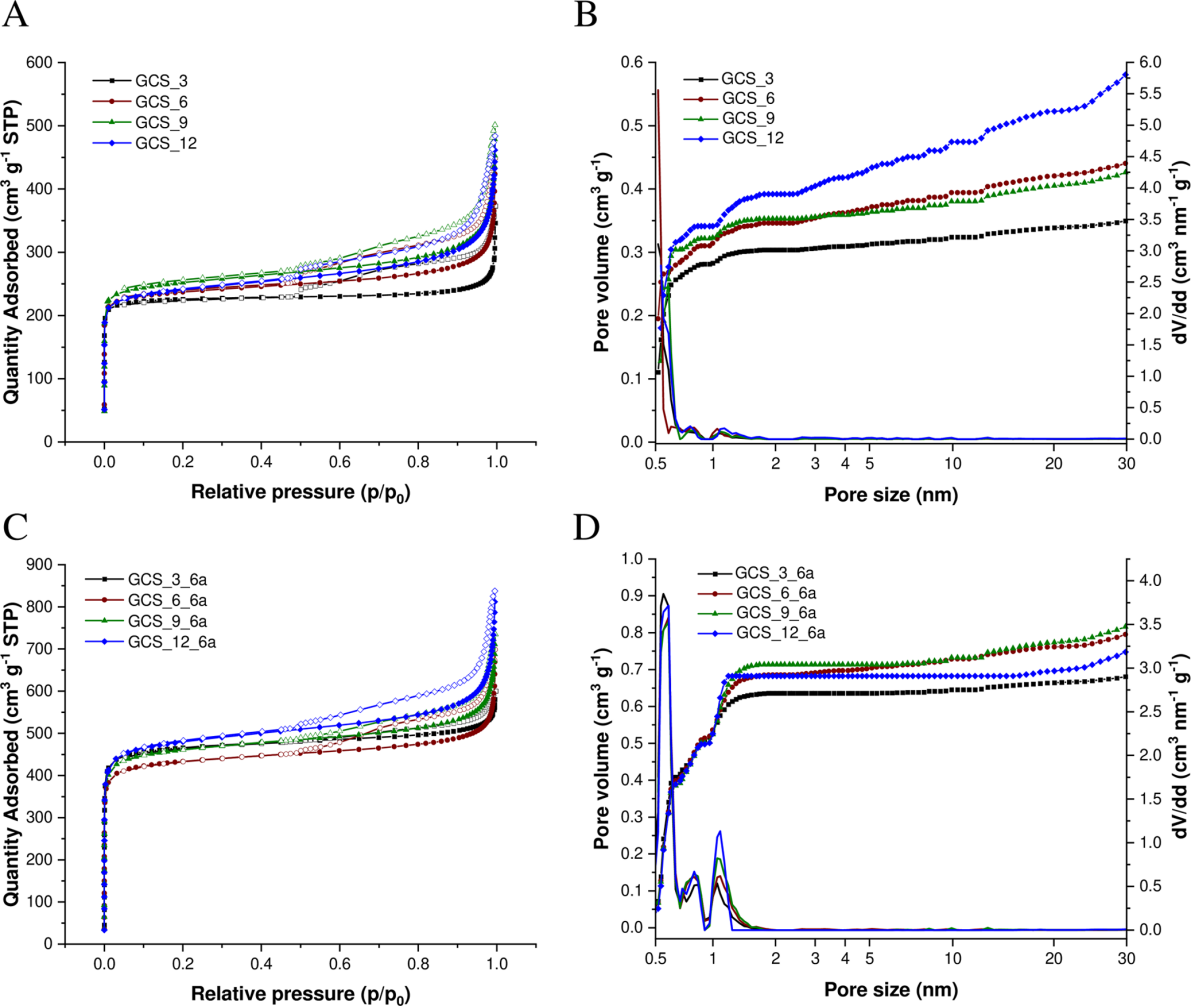
Nitrogen adsorption–desorption isotherms at −196 °C (A) and corresponding cumulative PSDs (symbols, left axis) and differential PSDs (lines, right axis); (B) of carbon spherogels prepared with PS concentrations of 3 wt % (black), 6 wt % (red), 9 wt % (green), and 12 wt % (blue) as well as for the activated carbon spherogels (C and D, respectively). Specific surface areas and PSDs were evaluated using NLDFT (N2 @ 77 on Carbon Slit Pores).

In order to analyze the pore structure of samples further, nitrogen sorption isotherms were recorded and are shown in Figure 7. All isotherms are of type IV and feature a significant H4 hysteresis according to IUPAC classifications.^48^ Based on these isotherms and by using the NLDFT and the assumption of slit pore geometry, the SSA, the total pore volume, and the micropore volume were calculated (Table 1), 850–895 m^2^ g^–1^, 0.35–0.59 cm^3^ g^–1^, and 0.30–0.39 cm^3^ g^–1^, respectively, for the non-activated carbon spherogels (GCS_3-12). A relevant micropore content, which is located in the carbon sphere walls, is indicated by a sharp nitrogen uptake at low pressures. These micropores themselves are generated during the carbonization step due to the evaporation of water, carbon dioxide, and hydrocarbons present in the organic gels. Consequently, the PS fragments evoked by thermal decomposition can evaporate.^18^ Furthermore, the hollow sphere interior volume is featured by the presence of a significant H4 hysteresis during desorption, which correlates with the hollow macroporous core enclosed by a microporous carbon shell. Moreover, the sharp nitrogen uptake at high relative pressures close to a *p*/*p*_0_ of 1 indicates the filling of the roughly 193 nm sized, hollow interior. Furthermore, the H4 hysteresis loop suggests ink-bottle-shaped pores, and the characteristic step down in the desorption branch at a relative pressure *p*/*p*_0_ of 0.5 indicates the occurrence of cavitation-induced evaporation due to the tensile stress limit of the condensed fluid inside the pores.^49−51^ Moreover, a distinct increase in the specific surface area of up to roughly 1970 m^2^ g^–1^ and in particular an increasing micropore volume of up to 0.71 cm^3^ g^–1^ are obtained after physically activating the carbon spherogels with CO_2_ for 6 h (GCS_3-12_6a). Precisely, micropores are generated during this process, indicated by the increase of adsorbed nitrogen at lower relative pressures in the nitrogen isotherms of the activated carbon spherogels (Figure 7C) as well as visualized by the their pore size distribution (PSD) (Figure 7D). In general, the activation duration has a critical influence on these parameters as a shorter activation time (4 h) yields lower specific surface areas and micropore volumes (Supporting Information, Figure S8 and Table S3).

### Electrochemical Testing

**Figure 8:**
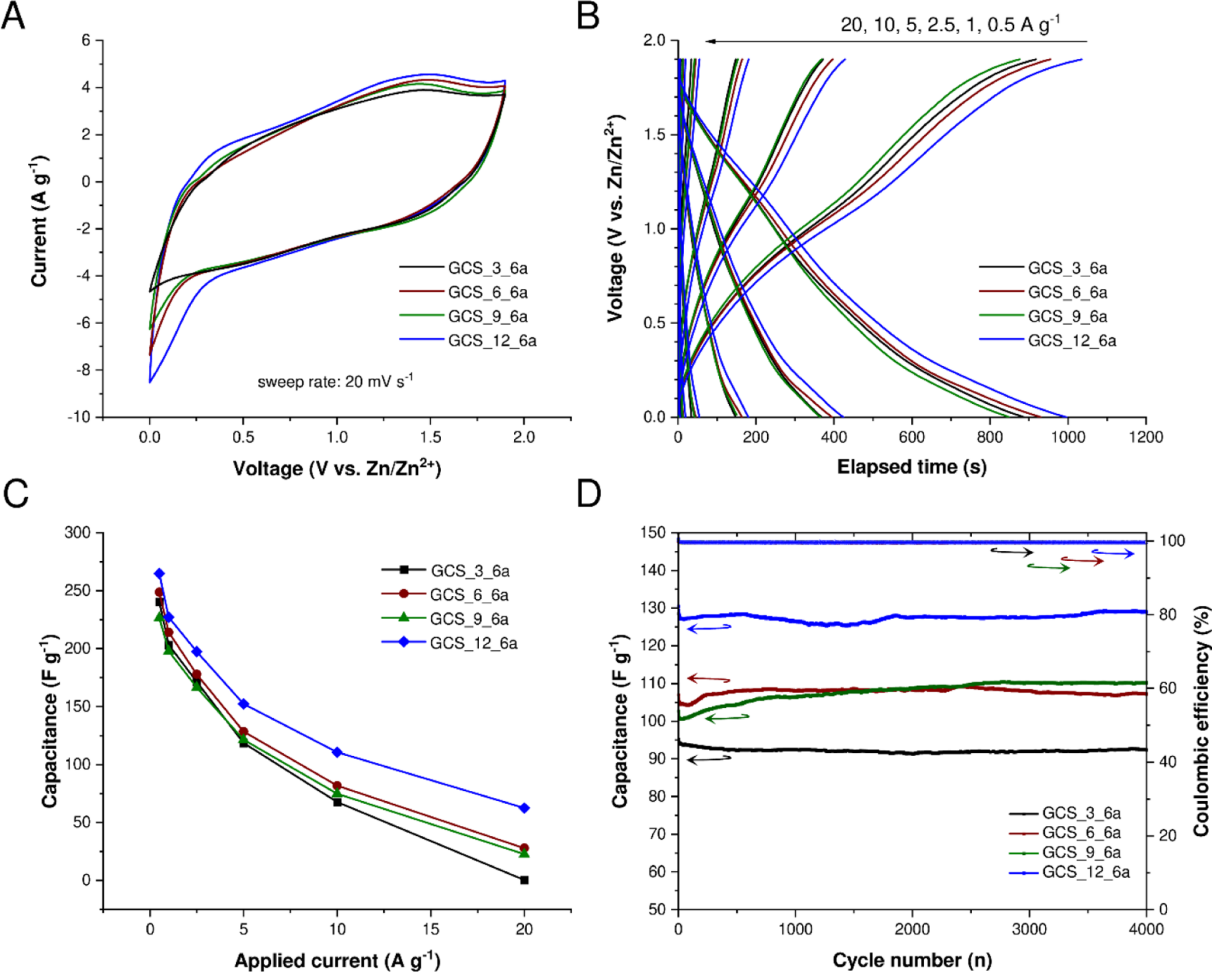
Electrochemical testing of the activated carbon spherogel samples in a zinc hybrid supercapacitor setup. GCS_3_3a (black), GCS_6_6a (red), GCS_9_6a (green), and GCS_12_6a (blue); cyclic voltammograms at a sweep rate of 20 mV s^–1^ (A); charge/discharge curves of GCS samples at different currents per gram (B); obtained specific capacitance (F g^–1^) dependent on applied current (C); and cycling stability at 8 A g^–1^ over 4000 cycles (D).

Tannin-derived ordered mesoporous carbons or porous carbons derived from chemically activated graphene have recently been reported to be impressive materials for supercapacitor applications.^52,53^ Therefore, the carbon spherogels synthesized at a pH of 1, with a T/W ratio of 0.05, and using different PS concentrations, were tested regarding their electrochemical performance and evaluated concerning their applicability as an electrode material for electric double-layer capacitors. Materials with higher specific surface areas tend to show better electrochemical performances, as they promote the diffusion of electroactive species.^52^ Hence, solely the 6 h CO_2_-activated carbon spherogels, prepared at different PS concentrations (GCS_3–12%_6a), with surface areas ranging from 1894 to 1971 g m^–2^ and micropore volumes of 0.68–0.82 cm^3^ g^–1^ (Figure 7C,D and Table 1), were evaluated as electrode materials for a hybrid supercapacitor with an aqueous electrolyte (3 mol kg^–1^ Zn(OTf)_2_) versus zinc. The hybrid supercapacitor setup with zinc as the anode material was chosen due to its high specific capacity, stability in aqueous electrolyte, and in particular natural abundance.^53^ Furthermore, the electrode preparation included using an aqueous-based slurry, a fluorine-free binder, and a mixture of Na-CMC and SBR.^54^Figure 8 shows the results of CV at 20 mV s^–1^, galvanostatic charge/discharge curves at various currents, specific capacitances dependent on applied currents, and long-term stability tests (4000 cycles at 8 A g^–1^).

The CV curves, obtained at a 20 mV s^–1^ sweep rate (Figure 8A), show a similar behavior for all the four investigated activated GCS materials in the oxidative region (positive current). A certain onset at 0.25 V is present in the negative current range, leading to more negative currents for the samples GCS_6–12_6a compared to GCS_3_6a. Such electrochemical behavior in carbon-based zinc hybrid supercapacitors has been previously reported and is typical at low potentials versus Zn/Zn^2+^.^53^ The charge/discharge profiles (Figure 8B) at different currents show again a similar behavior. The curves are almost linear in shape, as expected from CV. At currents of 0.5 A g^–1^, a slight overpotential is present, but it does decrease with the increase of charge/discharge currents. The obtained specific capacitances are presented in Figure 8C. The sample GCS_12_6a shows the best results compared to the other three GCS compounds, for example, achieving 265 F g^–1^ at 0.5 A g^–1^. With increased applied current, the specific capacitances decrease. In the case of GCS_12_6a, the capacitances decrease from 227 to 62 F g^–1^ from 1 to 20 A g^–1^, respectively. These results are in good agreement with previous results obtained from other activated carbons/graphene oxide zinc hybrid energy storage systems (Table S4).^53,55,56^ Hence, to demonstrate the feasibility of GCS materials, long-term tests were carried out (see Figure 8D). All compounds show a stable behavior over 4000 cycles, whereas the Coulombic efficiency, defined as the discharge capacity divided by the charge capacity, remains at 100% after 4000 cyles.^57^ GCS_12_6a shows the highest capacitance compared to the GCS_6-9_6a compounds, with a mean capacitance of 125 F g^–1^ over 4000 cycles at a current density of 8 A g^–1^. In all cases, the sample GCS_12_6a shows the best electrochemical properties and is a promising candidate for further electrochemical investigations as a sustainable electrode material for zinc hybrid supercapacitors or other metal-ion battery setups. Generally, it can be concluded that carbon spherogels with thinner wall thickness and higher specific surface areas yield the best electrochemical outcome in a hybrid zinc-based energy storage device. To further visualize the potential of the synthesized tannin-based carbon spherogels as electrode materials in a zinc hybrid supercapacitor setup, the charging and discharging process of the supercap, resulting in lighting of a red light-emitting diode (LED), is depicted in Figure S9 in the Supporting Information. However, overall it has to be acknowledged that solely first electrochemical results are presented for the hollow tannin-based carbon spheres, and an increment of electrochemical capacitance, for example, by doping the spheres, will be further investigated in the upcoming studies.

## Conclusions

In this study, we successfully presented a synthesis route toward sustainable, nanoscale carbon spherogels generated via PS templating and sol-gel processing of tannin and 5-HMF. Homogeneous, monolithic GCSs, consisting of a hollow carbon sphere with a microporous carbon shell, have been generated, as affirmed by SEM, TEM, and SAXS measurements. However, their generation requires a defined parameter setting, most importantly including the dilution ratio (T/W ratio) and the initial pH value. More precisely, varying these process parameters yields different carbon morphologies ranging from spherogel structures to aerogel-related structures, comprising a nanoparticle network.

The presented methodology shows great potential toward the preparation of sustainable, nanoporous, high surface area carbon materials due to several benefits: (1) the use of low-price, natural tree extract tannin, which is a waste product in the paper industry, as well as the use of the biomass-derived crosslinker 5-HMF instead of the commonly employed toxic formaldehyde, (2) mild and aqueous reaction conditions, (3) straightforward experimental setup, and (4) simple structure design, by, for example, altering the PS concentration or the duration of the carbon dioxide activation.

Moreover, these GCSs show a comparable electrochemical behavior to petroleum-based state-of-the-art carbons. Hence, these sustainable and novel materials represent an excellent alternative to the carbons prepared from environmentally harmful precursors, in regard to their applicability as electrode materials for zinc hybrid supercapacitors.

Overall, the preparation of a hollow carbon material on the basis of sustainable organic precursors with beneficial applicability in the field of energy storage is demonstrated. However, to further increase the sustainability of the synthesis, ongoing studies will focus on the use of a green template method and its feasibility to replace the utilized PS spheres.

## Supporting Information Available

SEM image of the used PS spheres; nomenclature of the synthesized gels; particle size distribution and zeta potential of PS spheres; TGA in the range of 20–1000 °C under an argon atmosphere: tannin/5-HMF aerogel without PS templating as reference, carbon spherogels, and dried PS spheres; scanning electron micrographs of the carbonized PS-templated tannin/5-HMF carbon gels at various pH values; Raman spectra of carbon spherogels with different PS concentrations; cumulative Raman fit of the carbon spherogels with different PS concentrations by deconvolution of the observed D- and G-bands into D*; nitrogen adsorption–desorption isotherms at −196 °C and cumulative PSDs and differential PSDs of the 4 h carbon dioxide activated carbon spherogels with different PS concentrations; charging and discharging process, resulting in lighting a red LED, of the GCS material in a hybrid zinc-based energy storage system; specific amounts employed for the generation of sustainable carbon spherogel samples; characteristic Raman data of carbon spherogels with different PS concentrations; physical characteristics of the carbon dioxide activated (4 h) carbon spherogel samples with varying PS template concentrations; and comparison with other carbon/graphite//zinc-based energy storage devices (PDF)
Supercapacitor charging and energy release via a propeller setup (MP4)
