# Supplementary material for: Tannin-Based Nanoscale Carbon Spherogels as Electrodes for Electrochemical Applications
Source: ACS Appl Nano Mater. 2021 Dec 2;4(12):14115–25. doi: 10.1021/acsanm.1c03431 (PMC8713360; doi:10.1021/acsanm.1c03431)
Supplement: Supplementary file 1 — an1c03431_si_001.pdf [file an1c03431_si_001.pdf]

Supporting Information

Tannin-based Nanoscale Carbon Spherogels as Electrodes for Electrochemical Applications

Ann-Kathrin Koopmann[a,b], Jorge Torres-Rodriguez[a,b], Miralem Salihovic[a], Juergen Schoiber[a], Maurizio Musso[a], Gerhard Fritz-Popovski[c], Nicola Huesing[a,b], Michael S. Elsaesser[a]

[a] *Paris-Lodron-University of Salzburg, Department of Chemistry and Physics of Materials, 5020 Salzburg, Austria*

[b] *Salzburg Centre for Smart Materials, 5020 Salzburg, Austria*

[c] *Montanuniversitaet Leoben, Institute of Physics, 8700 Leoben*

Corresponding author: michael.elsaesser@sbg.ac.at

Tel.: +43-662-8044-6262

Number of pages: 9
Number of figures: 9
Number of tables: 4

Figures:

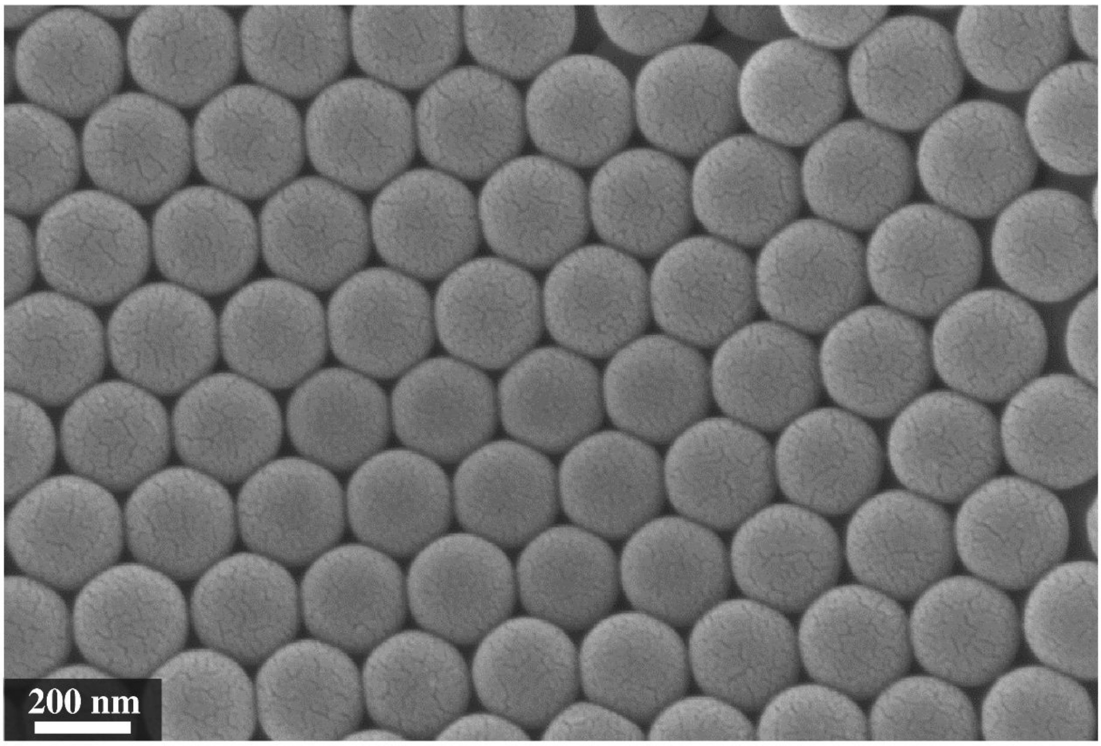

Figure S1. SEM image of the used PS spheres (average diameter size 213.0 nm ± 3.5 nm), determined by measuring 15 spheres using the SmartTiffV3 software. For sample preparation, a solution droplet was deposited on carbon tape, dried and sputtered with a gold film to avoid charging effects.

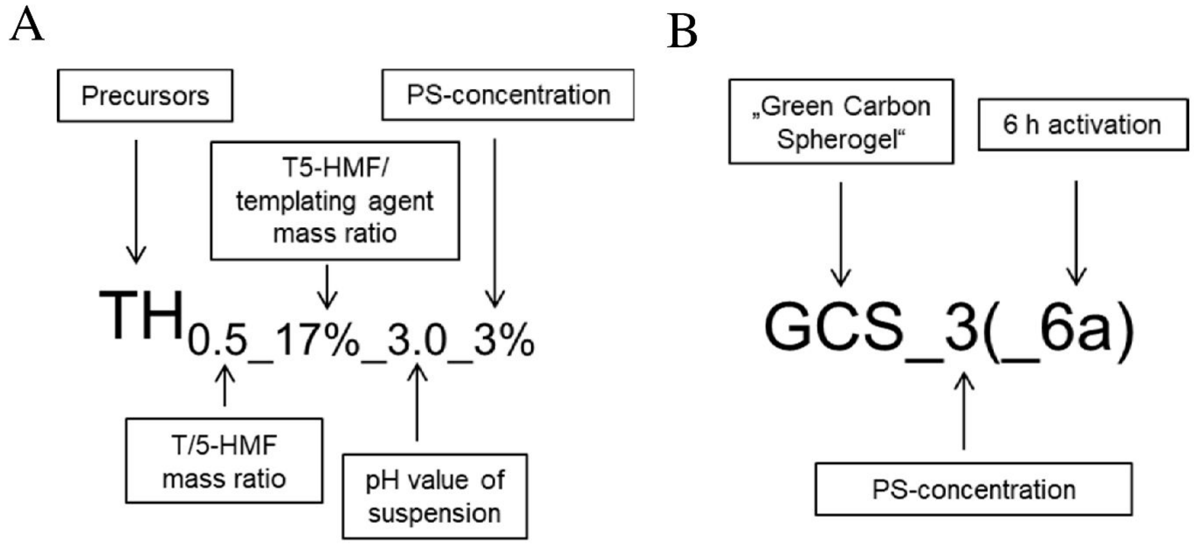

Figure S2. Nomenclature of the synthesized gels for preliminary tests (A) as well as of the synthesized carbon spherogels, which were characterized regarding their physical and chemical properties (B).

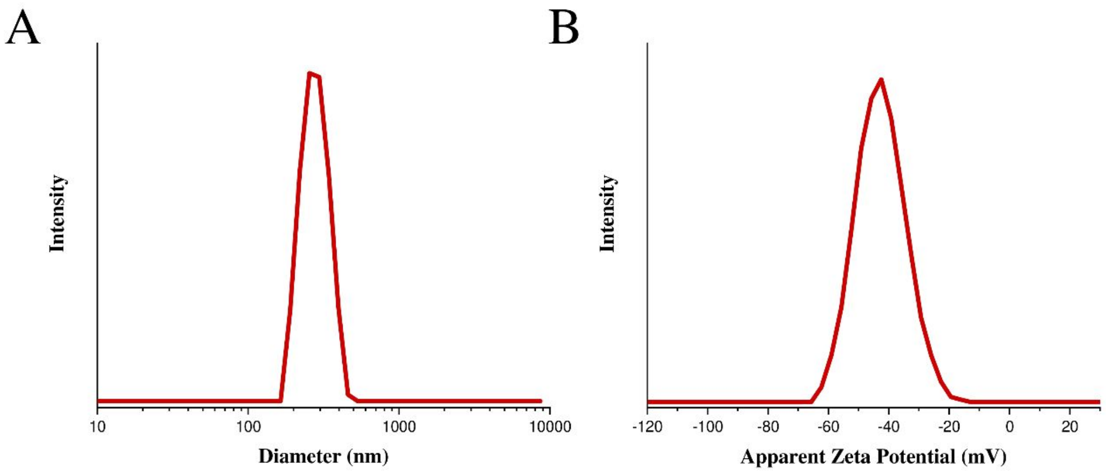

Figure S3. Particle size distribution of PS spheres determined by dynamic light scattering with an average hydrodynamic diameter of 272 nm (PdI = 0.011) (A); Zeta potential measurement of PS spheres showing an average value of -42.7 mV (B).

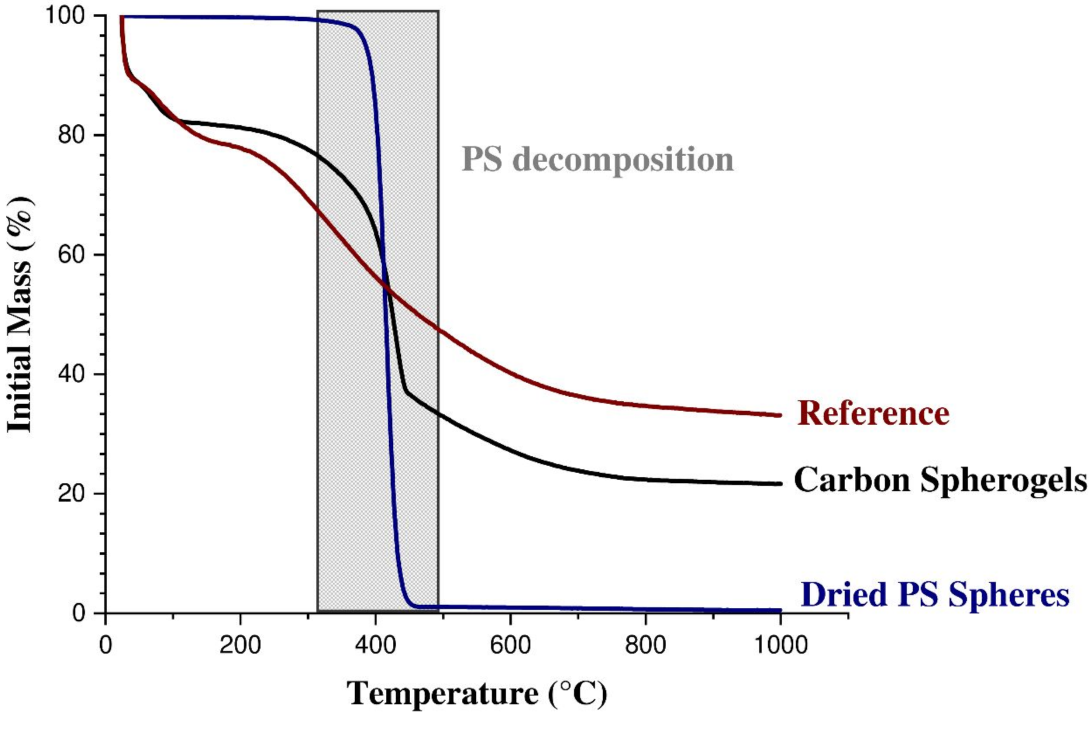

Figure S4. Thermogravimetric analysis (TGA) in the range of 20 to 1000 °C under argon atmosphere: Tannin/5-HMF aerogel without PS templating as reference (red); Carbonization process (PS template) to carbon spherogels (black) and dried polystyrene spheres (blue) with resulting residual masses of 29%, 22% and 0%, respectively. PS degradation is indicated by the grey box from 300 to 450 °C.

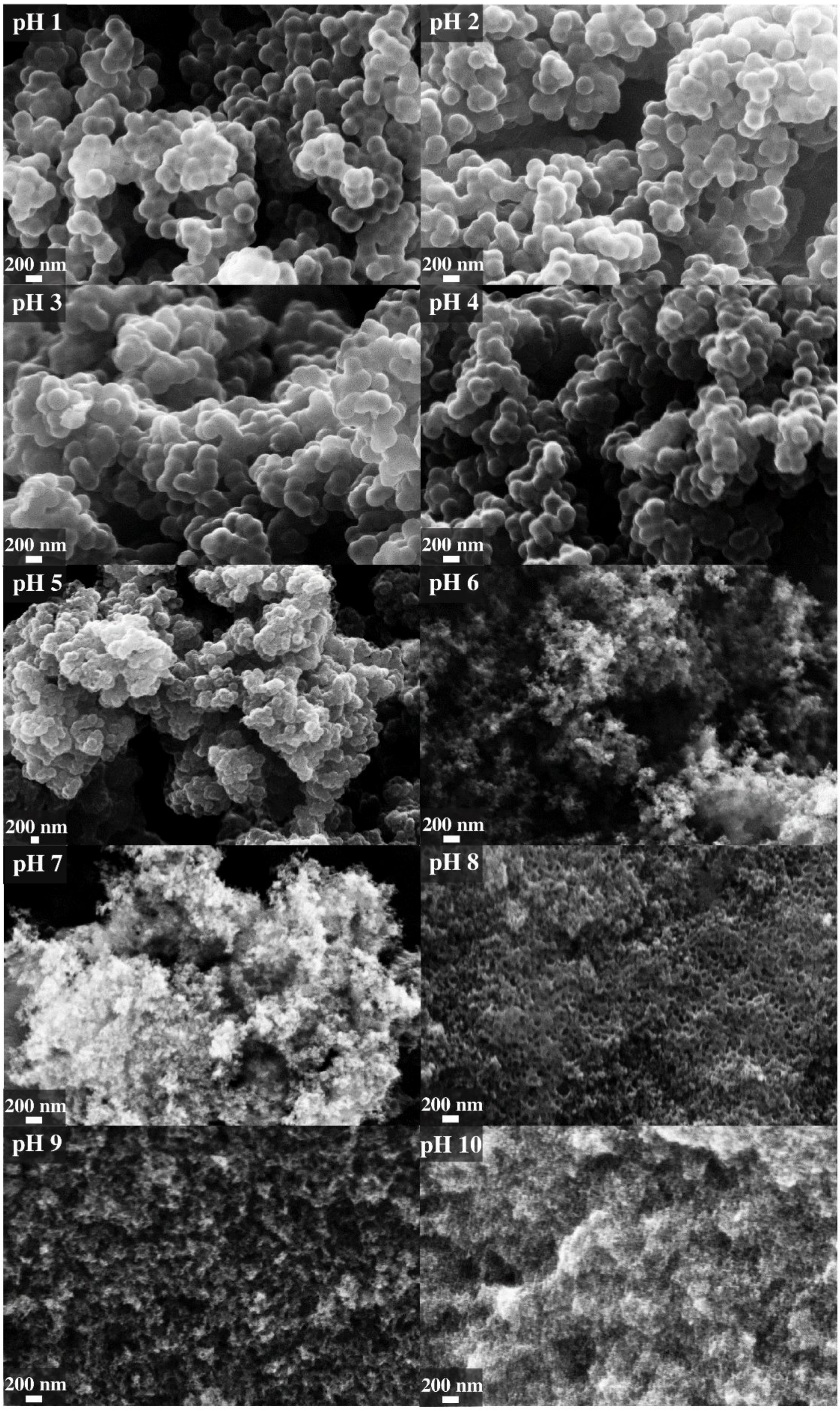

Figure S5. Scanning electron micrographs of the carbonized PS templated tannin/5-HMF carbon gels, generated at a T/W ratio of 0.05, a T/5-HMF ratio of 0.5 and at various pH values (1-10).

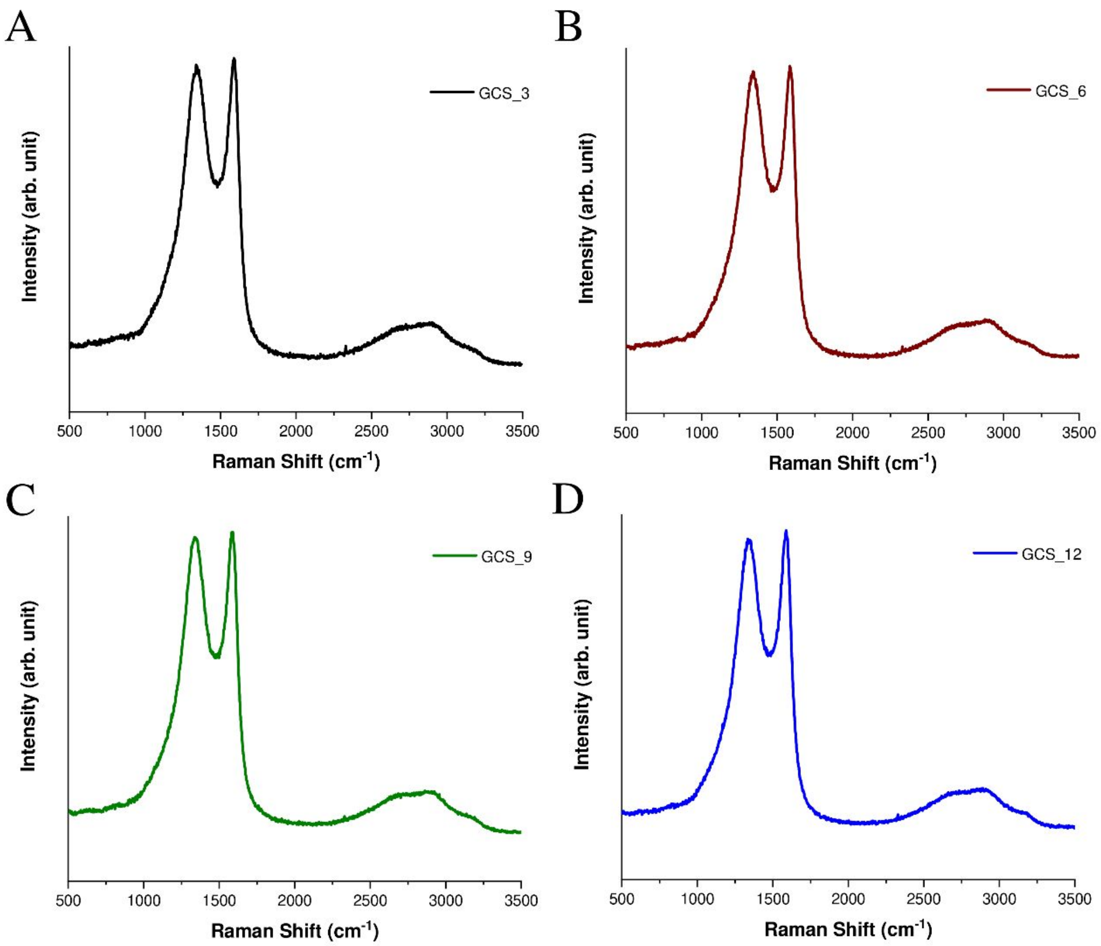

Figure S6. Raman spectra of carbon spherogels with different PS concentrations of 3 (black), 6 (red), 9 (green) and 12 wt% (blue), recorded by utilizing a 532 nm laser wavelength and a laser power of 4 mW.

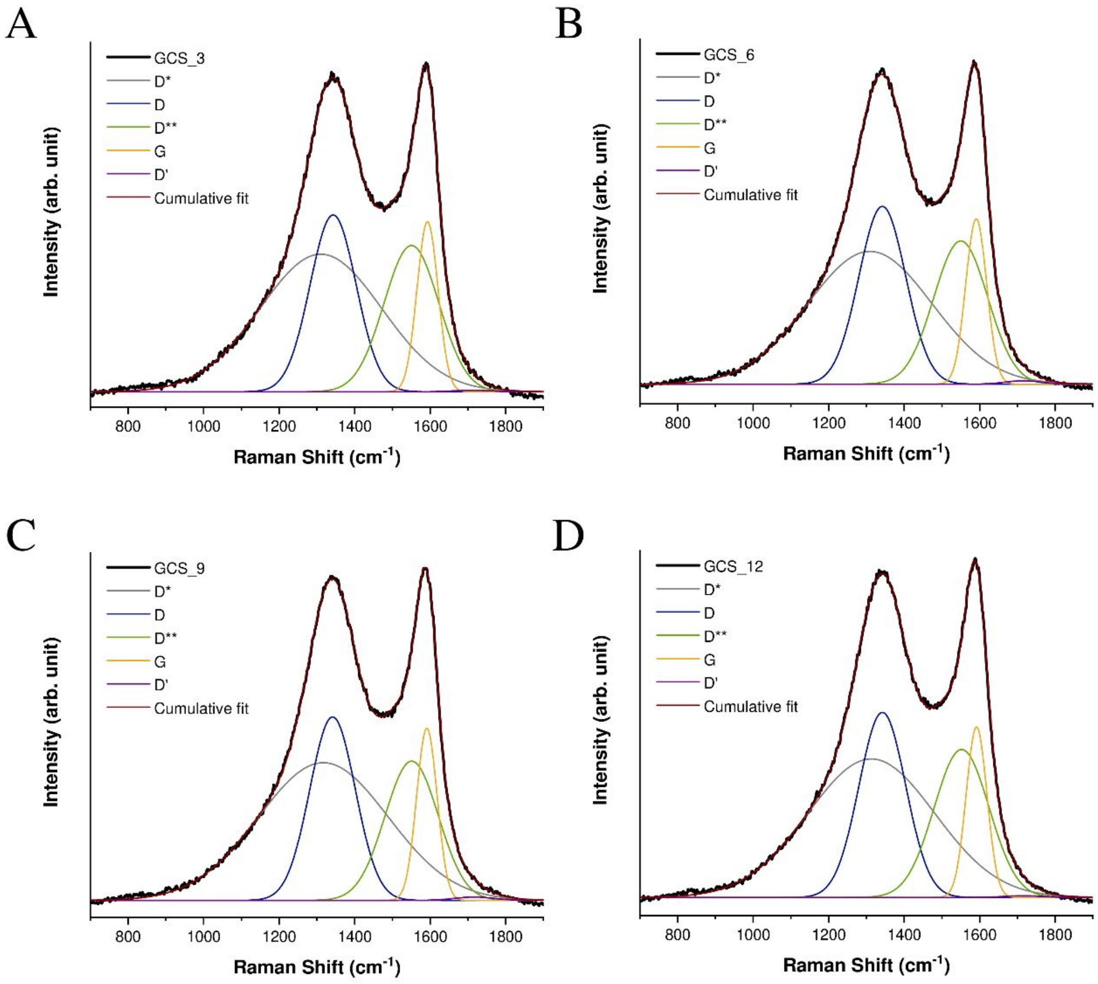

Figure S7. Cumulative Raman fit (red) of the carbon spherogels with different PS concentrations of 3, 6, 9 and 12 wt% (A, B, C, D, respectively) by deconvolution of the observed D- and G-bands into D* (grey), D (blue), D** (green), G (yellow) and D' (purple).

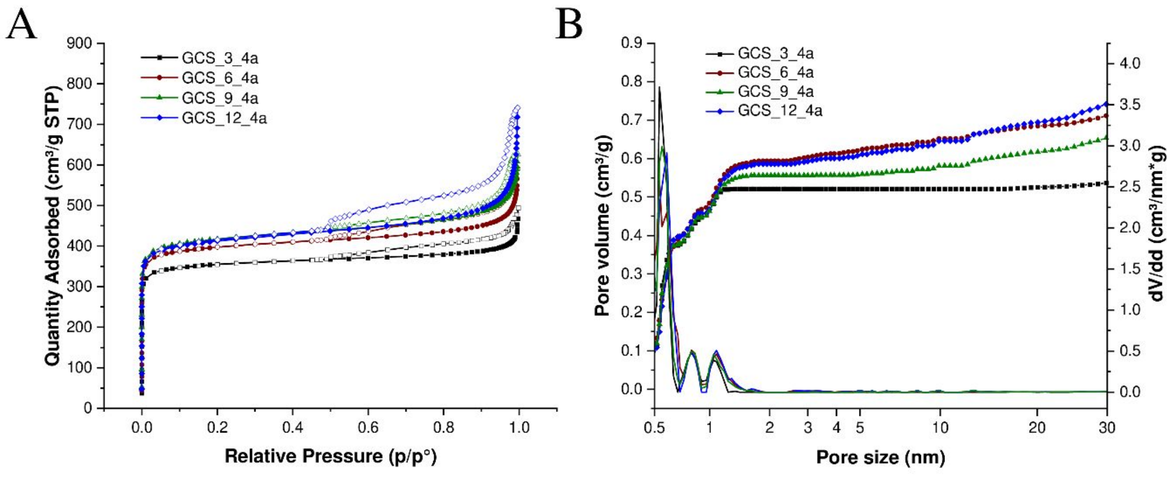

Figure S8. Nitrogen adsorption-desorption isotherms at -196°C (A) and cumulative pore size distributions (symbols, left axis) and differential pore size distributions (lines, right axis) (B) of the 4 hours carbon dioxide activated carbon spherogels with different PS concentrations (3, 6, 9 and 12 wt%). Specific surface areas and pore size distributions were calculated using a NLDFT model (N2 @ 77 on Carbon Slit Pores).

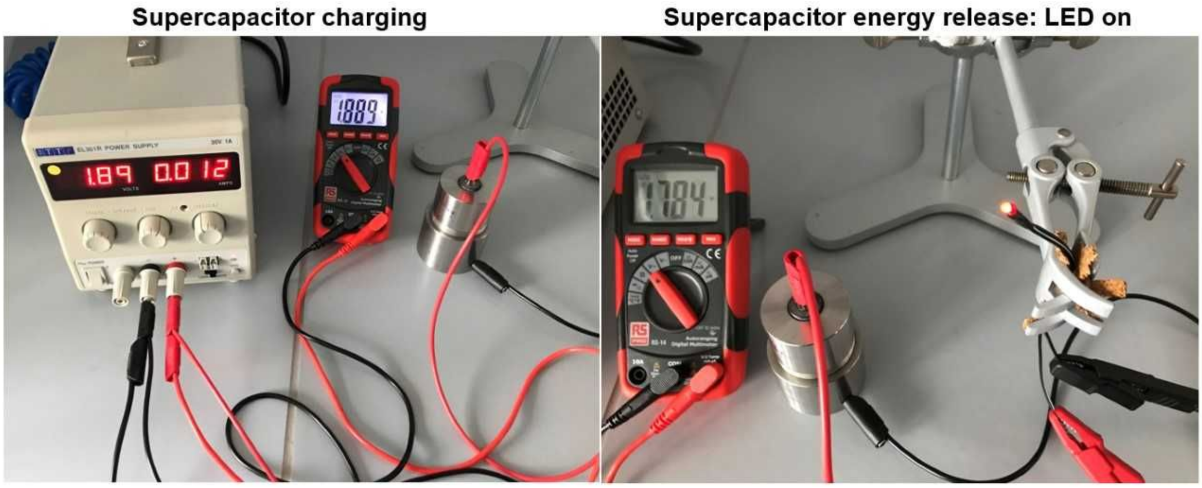

Figure S9. Charging and discharging process, resulting in lighting a red LED, of the green carbon spherogel material in a hybrid zinc-based energy storage system.

Tables:

Table S1. Specific amounts employed for the generation of sustainable carbon spherogel samples.

Sample	Tannin	5-HMF	PS solution				T/W ratio
			3 wt%	6 wt%	9 wt%	12 wt%	
	(g)	(g)	(g)	(g)	(g)	(g)	
**$TH_{0.5\_8\%\_X\_3\%}$**	0.13	0.26	4.77	-	-	-	0.03
**$TH_{0.5\_26\%\_X\_3\%}$**	0.50	1.00	5.75	-	-	-	0.09
**$TH_{0.5\_37\%\_X\_3\%}$**	0.63	1.25	5.10	-	-	-	0.12
**$TH_{0.5\_95\%\_X\_3\%}$**	1.25	2.50	3.95	-	-	-	0.32
**GCS_3**	0.31	0.63	5.68	-	-	-	0.05
**GCS_6**	0.31	0.63	-	5.69	-	-	0.05
**GCS_9**	0.31	0.63	-	-	5.70	-	0.05
**GCS_12**	0.31	0.63	-	-	-	5.71	0.05

Table S2: Characteristic Raman data of carbon spherogels with different PS concentrations of 3, 6, 9 and 12 wt%, listing the positions of the D- and G-band, the FWHM, and the peak area ratio A_D/A_G.

Sample	Band	Position (cm^{-1})	FWHM (cm^{-1})	A_D/A_G
**GSC_3**	D-band	1343	144	2.3
	G-band	1593	66	
**GSC_6**	D-band	1342	146	2.4
	G-band	1591	65	
**GSC_9**	D-band	1342	142	2.4
	G-band	1591	64	
**GSC_12**	D-band	1342	145	2.4
	G-band	1592	65	

Table S3. Physical characteristics of the carbon dioxide activated (4 h) carbon spherogel samples with varying PS template concentrations (3, 6, 9 and 12 wt%), obtained from nitrogen sorption. ($^a \leq$ 30 nm. $^b \leq$ 2 nm).

Sample	SSA (NLDFT) N2 @ 77 on Carbon Slit Pores by NLDFT (m²/g)	Specific pore volume^a (NLDFT) (cm³/g)	Micropore volume^b (NLDFT) (cm³/g)
**GCS_3_4a**	1697	0.54	0.52
**GCS_6_4a**	1707	0.72	0.59
**GCS_9_4a**	1737	0.65	0.57
**GCS_12_4a**	1790	0.75	0.59

Table S4. Comparison with other Carbon/Graphite // Zinc-based energy storage devices.

Energy storage system (Cathode//Electrolyte//Anode)	Operating voltage	Capacitance per weight	Capacitance per volumne	Reference
**aMEGO// 3b $Zn(OTf)_2$// Zn**	0 – 1.9 V	166 F g^{-1} at 0.5 A g^{-1}	/	Adv. Energy Mater., 2019, 9, 1902915
**AC (YEC-8A)// 2M $Zn(SO_4)$// Zn**	0.2 – 1.8 V	308 F g^{-1} at 0.5 A g^{-1}	/	ChemSusChem., 2021, 14, 1700-1709
**AC// 2M $Zn(SO_4)$// Zn**	0.5 – 1.5 V	259 F g^{-1} at 0.5 A g^{-1}	/	Adv. Mater., 2019, 31, 1806005
**GCS_3_6a// 3b $Zn(OTf)_2$// Zn**	0 – 1.9 V	240 F g^{-1} at 0.5 A g^{-1}	163 F cm^{-3} at 0.5 A g^{-1}	Present work
**GCS_6_6a// 3b $Zn(OTf)_2$// Zn**	0 – 1.9 V	249 F g^{-1} at 0.5 A g^{-1}	199 F cm^{-3} at 0.5 A g^{-1}	Present work
**GCS_9_6a// 3b $Zn(OTf)_2$// Zn**	0 – 1.9 V	227 F g^{-1} at 0.5 A g^{-1}	186 F cm^{-3} at 0.5 A g^{-1}	Present work
**GCS_12_6a// 3b $Zn(OTf)_2$// Zn**	0 – 1.9 V	265 F g^{-1} at 0.5 A g^{-1}	201 F cm^{-3} at 0.5 A g^{-1}	Present work
